# Tooth Repair and Regeneration

**DOI:** 10.1007/s40496-018-0196-9

**Published:** 2018-10-25

**Authors:** Ana Angelova Volponi, Lucia K. Zaugg, Vitor Neves, Yang Liu, Paul T. Sharpe

**Affiliations:** 1 Centre for Craniofacial and Regenerative Biology, Dental Institute, King’s College London, London, UK; 2 Department of Periodontology, Endodontology and Cariology, University Center for Dental Medicine Basel, University of Basel, Basel, Switzerland

**Keywords:** Regenerative dentistry, Dentinogenesis, Bio-tooth, Biological repair, Reparative dentin

## Abstract

**Purpose of Review:**

Current dental treatments are based on conservative approaches, using inorganic materials and appliances.

This report explores and discusses the newest achievements in the field of “regenerative dentistry,” based on the concept of biological repair as an alternative to the current conservative approach.

**Recent Findings:**

The review covers and critically analyzes three main approaches of tooth repair: the re-mineralization of the enamel, the biological repair of dentin, and whole tooth engineering.

**Summary:**

The development of a concept of biological repair based on the role of the Wnt signaling pathway in reparative dentin formation offers a new translational approach into development of future clinical dental treatments.

In the field of bio-tooth engineering, the current focus of the researchers remains the establishment of odontogenic cell-sources that would be viable and easily accessible for future bio-tooth engineering.

## Introduction

Tooth loss is a global health problem representing a burden to society and the economy [1]. It affects an individual’s capacity for biting, chewing, smiling, speaking, and psychosocial wellbeing. Complete loss of natural teeth is widespread, particularly affecting older people [2, 3]. Dental caries, periodontal disease, and genetic disorders are major causes of tooth loss.

Caries is reported as the most common disease worldwide [4, 5]. Current dental treatments used to replace missing tooth structure or missing teeth are based on conservative therapies such as fillings, made of inert dental materials, fixed dental bridges, or removable dentures and dental implants.

Osseointegrated dental implants revolutionized dentistry as they provide restoration of lost function without affecting healthy teeth [6]. Although current dental implants mark notable advantages in osseointegration and soft tissue adaptations, the concept of the treatment is based on the usage of inert materials in direct contact with bone tissue and absence of periodontal ligament (PDL) tissue. The PDL physiologically provides a buffered distribution of mastication forces and when absent can often lead to jaw bone resorption [7, 8].

*Regenerative dentistry is an emerging concept that challenges the modern dentistry to step up the dental research and translate the scientific knowledge into new future clinical treatments.*

*The approach is based on understanding the underlying mechanisms of tooth development and the biological processes of healing and repair, creating a solid knowledge of principles that could be applied in harnessing the natural healing potential of the dental tissues, or regenerating (engineering) the damaged tissue or organ.*

## Healing and Engineering Different Dental Tissues

### Biomimetic Approach in Repairing the Damaged Enamel

#### Repairing a Cell-Free Tissue

A tooth is a complex organ consisting of a soft connective tissue (dental pulp) encased in a chamber of differently mineralized hard tissues (enamel, cementum, and dentin). The outer mineralized tissue in the crown region, the enamel, is the highest mineralized tissue of the human body, characterized by an absence of cells. It provides the first hard barrier towards the outer environment, protecting the tooth from damage.

During tooth development, the ameloblasts, which are responsible for the formation of enamel, undergo programmed cell death at the maturation stage and no longer exist in the mature enamel [9].

Therefore, once damaged, enamel cannot be biologically regenerated/repaired. Hence, the concept of “healing” of the damaged enamel consists in repairing by acellular re-mineralization. Traditionally, fluoride (F) and calcium phosphate nanocrystals are applied to re-mineralize the eroded enamel matrix and act by inhibiting demineralization by fluoride incorporation in the crystal lattice, resulting in lower solubility of enamel [10], and having a potential to protect the outer ~ 30 μm of the tooth [11]. The newly formed hydroxyapatite usually lacks the structure and mechanical properties of the natural enamel [12, 13]. Therefore the biggest challenge lies in recreating the hierarchical structure on the surface of the damaged enamel. The unique cross-arranged structure of enamel exhibits two important components: prismatic and interprismatic areas, which have different stabilities to resist acid erosion [14]. So, the challenge remains to synthesize apatite nanocrystals with a proper oriented structure, similar to the natural enamel, directly on the surface of the damaged enamel and in an oral environment [15, 16].

**Fig. 1:**
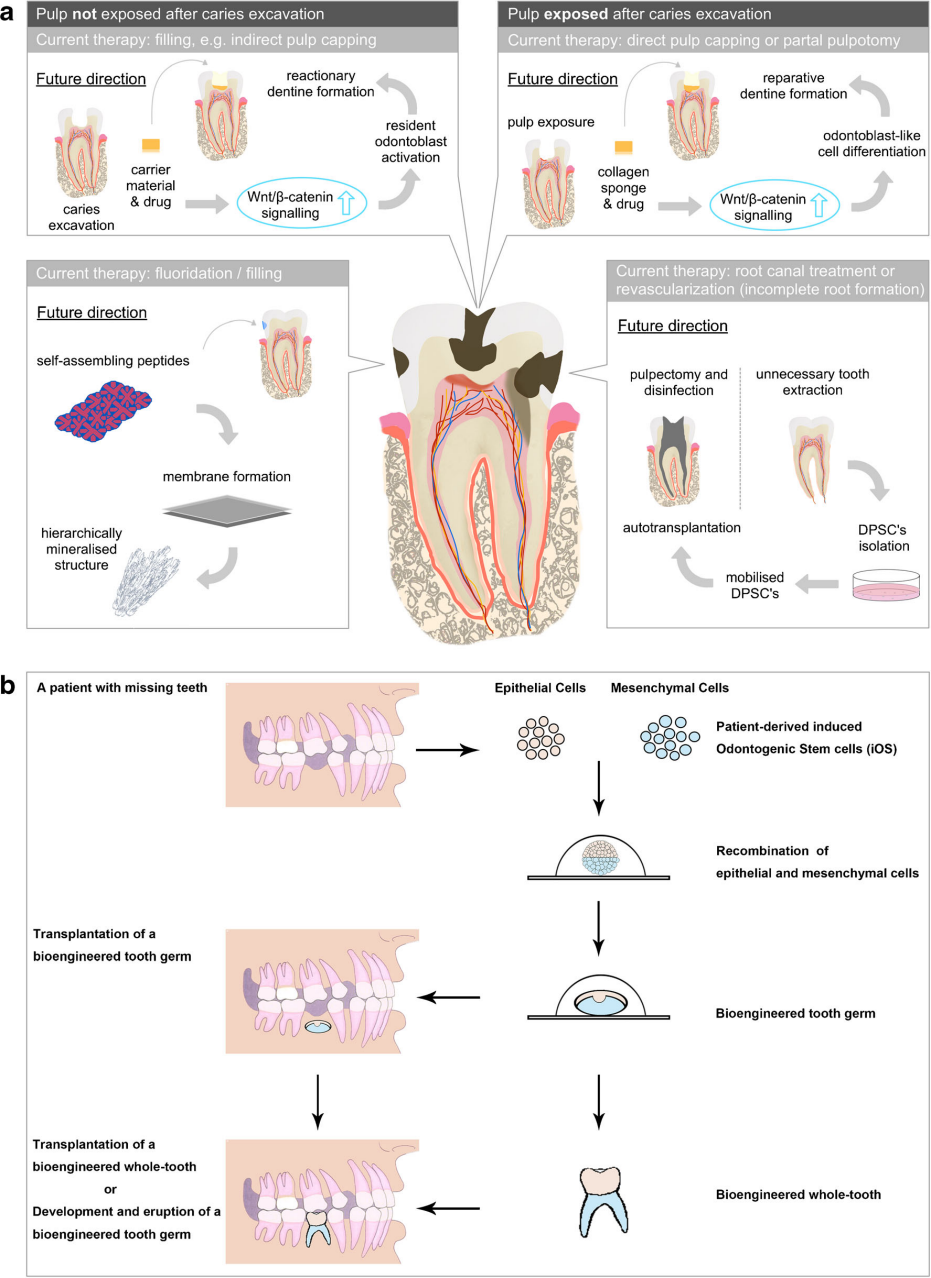
Schematic representation of different approaches for dentine-pulp complex repair/regeneration (**a**) and stem-cell-based whole-tooth bioengineering (**b**). **a** Pharmacological modulation of Wnt/β-catenin signaling pathway shows natural dentine apposition in both, deep cavitation without pulp exposure (reactionary dentine, upper left box) and cavitation with exposed pulp tissue (reparative dentine, upper right box), as long as the underlying pulp tissue is vital and harbors resident odontoblasts and dental pulp stem cells (DPSCs) respectively. The simple applicability of this technique by using a drug-enriched collagen sponge makes it ideal for a translational clinical treatment approaches. In case of pulp infection and necrosis (lower right box), current therapies include orthograde root canal treatment or, in selected cases with incomplete root formation, revascularization procedures. Recent cell-based approaches show that autologous isolated, expanded, and mobilized DPSCs have the capacity to re-innervate (positive response on pulp testing) a pulpectomized and disinfected tooth after auto-transplantation; however, this approach is highly technique sensitive and might remain in facilities with specialized equipment and laboratories for selected cases only. Non-cell-based approaches for mimicking lost enamel-structure exists (lower left box) yet the mineralization potential of self-assembling peptides needs to be further evolved and clinically tested. **b** Suitable adult sources of epithelial and mesenchymal cells are collected from the patients with missing teeth and expanded in vitro. Either epithelial or mesenchymal cell populations are induced to be odontogenic (capable of initiation of de novo odontogenesis) and recombined with the responsive cell population counterpart. An early-stage tooth primordium can be generated from the epithelial-mesenchymal-cell recombination, which can be subsequently either directly transplanted at the location of the missing tooth or cultured ex vivo to form a whole tooth to replace the missing tooth

Recently, different biomimetic approaches have been developed to synthesize artificial dental enamel. In a study by Kind L. et al. [17], self assembling peptides were used to facilitate the subsurface mineralization of the enamel in carious lesion, while other groups used elastin-like polypeptide-assisted biomimetic approach to synthesize artificial dental enamel (Fig. 1a) [16]. Although these materials act only on the surface of enamel demineralization at present, enamel-oriented growth sheds a light on the potential of structural and mechanical regeneration of enamel cavitation.

Still, a major goal in the fast-developing material science remains creation, design, and development of bioinspired functional structures, using synthetic hierarchical materials with enhanced functionality, like the dental enamel [18•]. Moreover, the open question remaining is the time needed for enamel remineralization to form a functional enamel tissue. This issue will lastly determine the viability of this approach for a clinical treatment.

### Biological Repair of the Dentine-Pulp Complex

The dental pulp is a soft connective tissue, containing different cell populations, which is encased in a thick, porous mineralized chamber. As a clinical approach, keeping the vitality of the dental pulp has always been the goal for a successful long-term restorative dental treatment. In the adult pulp, cell division and the secretory activity of odontoblasts decrease in comparison to the developing stage; however, these processes are re-activated following pulp damage.

In shallow enamel and enamel/dentinal damage, odontoblasts can survive, and its activation supports repair, protecting the dental pulp via reactionary dentine formation [19, 20]. However, in situations of deep cavitation or trauma that involves dental pulp exposure, odontoblasts may not survive and will have to be replaced, requiring a cascade of stem cell activation, proliferation, and differentiation into new odontoblast-like cells that will culminate into reparative dentine secretion [21]. Because of the capacity of teeth to repair themselves and their accessibility, Gronthos and collaborators [22] researched and identified a population of new cells isolated from the dental pulp of human third molars. They termed these cells dental pulp stem cells (DPSCs) and showed that these cells produce dentine in vitro [23]. Although these data show that dental pulp cells have the capacity to repair damaged dental tissues, when it comes to in vivo tooth repair, the dental reparative capacity is limited to the critical size of the damage [24, 25]. In large, exposed pulpal injuries, the pulp is exposed to microorganisms from the oral cavity, and if the infected dentine is not completely removed before applying a biocompatible material while directly capping the exposed pulp, the dental pulp will undergo necrosis. This highlights the clear need to generate a therapy that stimulates the full biological potential the dental pulp has to repair injuries comprising dentine and pulp.

### Reactionary Dentin

During primary and secondary dentin deposition, odontoblasts secrete growth factors and proteins that are fossilized in the dentine matrix after mineral maturation [26]. As decay demineralizes the dentine, these growth factors and proteins are released to the dental pulp to activate odontoblasts, immune cells, and organize the reactionary dentine matrix secretion [27]. Hydraulic silicate cements, e.g., mineral trioxide aggregate (MTA), Biodentine, total fill, endosequence, calcium-enriched cement etc., claim to be bioactive. As demonstrated by Loison-Robert and colleagues [28], Biodentine promotes mineralization when in contact with dental pulp stem cells (DPSCs); however, it does not affect DPSC proliferation. Interestingly, the only biological factor that Biodentine has shown to increase is TGF-β1 [29]; however, Neves and Sharpe [30••] showed that TGF-β and BMP are not pivotal for reactionary dentine formation but for tubular organization of reactionary dentine secretion. Reactionary dentine secretion is affected, however, by changes in the Wnt pathways. Wnt activation via GSK-3 inhibitor drugs increased reactionary dentine secretion, although Wnt inhibition did not impair reactionary dentine secretion (Fig. 1a) [30••].

One clinical specialty that could benefit from the advances of a reactionary dentine promoting material is prosthetic dentistry. The creation of a material that cements a crown and stimulates reactionary dentine formation might change the prosthetic planning approach, as it increases the rate of pulp vitality post tooth preparation to receive a crown. Biomaterials with the capacity to incorporate Wnt activators, such as Bioglass, have been developed [31], but further testing is still to be done.

### Reparative Dentine

When a dental injury reaches the dental pulp, a more complex clinical and biological approach is taken to preserve pulp vitality and reconstruct the lost dentine. Studies have revealed that dentine matrix derivatives and breakdown products from the dental pulp influence pulp cell migration. Recruited cells exhibited increased stem cell marker expression indicating that dental ECMs and their breakdown products selectively attract progenitor cells that contribute to repair processes [25, 32]. These mobilized resident dental pulp mesenchymal stem cells differentiate into new odontoblast-like cells that secrete a form of tertiary (reparative) dentine [33•, 34•, 35–37]. This dentine is laid in a form of a thin band of dentine (dentine bridge) that walls off the pulp from bacterial infection. Studies have focused in understanding the underlying mechanisms of this process of “natural healing,” in order to learn how to pharmacologically trigger this event for promoting reparative dentine formation [34•].

A recent preclinical study showed that small molecules delivered via a biodegradable collagen sponge were able to provide an effective repair of experimentally induced deep dental lesions by stimulating Wnt/β-catenin signaling and hence promote reparative dentine formation [38]. This study was based on the natural underlying mechanisms and pathways that are pivotal for the healing mechanisms of the dental pulp. The activation of Wnt/β-catenin signaling is an immediate early response to tissue damage; moreover, Wnt receiving cells become odontoblast-like cells, proving they are stem cells [34•, 38–42]. After confirming that Wnt/β-catenin is upregulated following tooth damage, the study showed that an addition of small-molecule Wnt agonists, tested in human clinical trials for Alzheimer, can stimulate the dental natural response, triggering reparative dentine formation and thus restoring the lost dentine structure with naturally generated new dentine. The striking “simplicity” of this technique makes it ideal for a translatable clinical technique; also, it helps opening a new door for more “biomimetic” approaches aiming to repair the dentine-pulp complex naturally (Fig. 1a). The fact that these particular small-molecule Wnt agonists are undergoing clinical trials already contributes further to the potential of translation into future dental treatments through an adequate incorporation into a dental material as a carrier.

When microorganisms or their endotoxins reach the dental pulp and it is diagnosed with irreversible pulpitis, because this condition cannot be reversed, the common treatment for this situation has been pulpectomy, regardless of the amount of the remaining unaffected pulp tissue. With the standard protocol, the entire pulp has to be removed, followed by root canal treatment, disinfecting the pulp space and replacing it with inorganic materials [43]. However, new approaches on de novo regeneration of the dental pulp have been investigated and will be further discussed below.

### De Novo Regeneration of Dental Pulp

An approach for repairing/regenerating the lost dental pulp tissue, following a root canal treatment is the “de novo” regeneration of dental pulp tissue. Currently, clinical procedures are postulated to successfully revascularize infected teeth with incomplete root formation to achieve full root length development and dentine thickness. A variety of promising cases have been published that indicated pulp revascularization with pulp- and dentin-like tissue formation and healing of apical inflammation [44–46]. However, this procedure is limited to infected or non-infected immature teeth with open apices [47]. To address tissue regeneration in fully formed teeth, procedures based on cellular approaches should be further considered and developed. Among the potential sources of stem cells isolated from teeth (DPSCs, stem cells from human exfoliated deciduous teeth [SHED] and stem cells of the apical papilla [SCAP]), Cordeiro and colleagues [48] suggested that SHED was the most valuable cell source for dental pulp tissue engineering due to its capacity to generate pulp tissue in human tooth slices with similar architecture to those of a physiologic dental pulp.

In 2010, Huang et al. [49] used different dental stem cells isolated from human dental pulp of permanent teeth (DPSCs) and stem cells isolated from the apical papilla region of human third molars (SCAP cells), to demonstrate de novo regeneration of dental pulp in empty root canal spaces. These cells were seeded onto poly-D,L-lactide/glycolide scaffold and inserted into the canal space of root fragments followed by subcutaneous transplantation into immunocompromised-SCID mice. After a period of 3 to 4 months, a histological analysis revealed that the root canal space was filled with pulp-like tissue with well-established vascularization. Moreover, a continuous layer of mineralized tissue resembling dentine was deposited on the existing dentinal walls of the canal. This dentine-like structure appeared to be produced by a newly formed layer of odontoblast-like cells [49].

In a more advanced attempt to regenerate whole dental pulp in a real clinical scenario, a recent clinical pilot study in humans assessed the therapeutic potential and safety of mobilized dental pulp stem cells (MDPSCs) to regenerate the dental pulp de novo in teeth that suffered from irreversible pulpitis without any periapical lesions [50, 51]. DPSCs were isolated from a small amount of pulp tissue of autologous discarded teeth using a granulocyte colony stimulating factor (G-CSF)-induced mobilization method. MDPSCs were successfully transplanted in previously disinfected, empty root canal spaces in vivo. Magnetic resonance imaging (MRI) and cone beam computed tomography (CBCT) revealed changes in the dental pulp indicating a pulp-like regenerated tissue and new dentine apposition after 24 weeks in most cases with positive response to electrical pulp testing (Fig. 1a) [50, 51].

While these studies focused on the usage of different dental stem cells in dental pulp tissue engineering, other studies focused on exploring new mechanical systems as options for scaffolds in pulp regeneration [52]. New injectable microsphere systems, where vascular endothelial growth factor (VEGF) binds with heparin and is encapsulated in heparin-conjugated gelatine nanospheres of a biodegradable poly L-lactic acid (PLLA) microsphere, mimic natural ECM-like collagen structures and hence act as carrier for DPSC leading to pulp tissue formation by promoting their proliferation/differentiation. Additionally, these systems provide a controlled release of the VEGF resulting in newly formed blood vessels within the regenerated tissue [52, 53].

Over the last decades, conservative endodontic procedures focused on techniques to enhance root canal disinfection and irrigation in order to address infection control of complex anatomical structures like isthmuses, and lateral canals or the apical delta. Since the presence of microbes in such areas can contribute to persistent inflammation, all procedures of de novo regeneration need to follow a procedure of complete root canal disinfection. From a clinical perspective, all the above-mentioned cellular-based cases of de novo pulp regeneration have been performed in teeth with relatively simple root canal anatomy and rather large canals. Hence, clinical applicability of teeth with more complex root canal anatomy may be questioned.

The idea of supporting the seeded dental stem cells, in this case dental pulp stem cells from adult teeth (DPSCs), providing a three-dimensional, controlled environment with added growth factors, emphasizes once more the underlying concept of the “regenerative” approach, where the biological system is mimicked and recreated in its complexity.

## Whole-Tooth Bioengineering

Whole-tooth bioengineering has always been the ultimate goal of regenerative dentistry. (Fig. 1b) Despite recent progress in this field [54–56, 57••, 58], we are still facing a number of difficult challenges to overcome. The basic principle of this “organ engineering” approach is understanding the mechanisms that regulate the embryonic tooth development and recreating these events in vitro, mimicking the natural cascade of signaling that occurs during organ formation [57••].

Due to its non-essential function and accessibility, it represents an important model to study organogenesis. In common with other ectodermal appendages, like hair follicles and exocrine glands (mammary, sweat, and salivary), tooth morphogenesis is guided by reciprocal interactions between epithelial and mesenchymal tissues and progresses through distinct stages [59, 60]. The knowledge gained in bio-tooth engineering potentially could have a broader impact in the field of regenerative medicine and the repair of different organs.

### Mimicking the Natural Events of Tooth Development

Several decades ago, classical tissue recombination experiments demonstrated sequential signaling between the dental epithelium and the mesenchyme of different origins and stages, where the epithelium acted as an inductive tissue [61, 62]. This odontogenic induction in the epithelium is lost early, as it naturally happens during the embryonic tooth development and then switches to mesenchyme. Thus, the mesenchyme becomes the odontogenic inductive source.

In 2003, Yamamoto and collaborators [63] showed the ability of embryonic tooth germ cells to re-aggregate following dissociation and form teeth. Other studies followed where epithelium and mesenchyme tissues from E14.5- to E12.5-stage mouse tooth germs were separated and the cells dissociated and recombined to form normal teeth [64–67].

This reciprocal tissue induction that takes place during the early stages of tooth development, whereby the epithelium first induces tooth formation in the mesenchyme followed by a reciprocal induction from mesenchyme to epithelium, has been utilized to suggest a basis for whole-tooth bioengineering that could employ adult cells [59, 68, 69].

In 2004, a study by Ohazama et al. [69] showed that when mesenchyme cells derived from adult bone marrow are combined with inductive-stage embryonic dental epithelium, tooth formation is induced, and the adult mesenchymal cells respond and fully contribute to tooth development [69]. Another study in 2013 [70] showed that human gingival epithelial cells were able to respond to the inductive signal of mouse tooth embryonic mesenchyme, resulting in formation of fully formed teeth.

However, in spite of the fact that adult stem cells can respond to an inductive odontogenic signal and participate in tooth formation, the only cells that have been shown to be capable of tooth-inductive capacity are odontogenic embryonic cells, derived from inductive embryonic tooth-germ tissue (epithelium or mesenchyme) [54–56, 57••, 58]. Furthermore, in all experiments reported to date, the inductive cells, whether epithelial or mesenchymal, do not retain their inductive capacity following in vitro expansion [71]. This defines one of the biggest challenges in the field of biotooth engineering, which is to identify adult cell populations that retain their odontogenic potential and can be expanded in large numbers. Moreover, these cells should ideally be allogeneic, where one population, either mesenchyme or epithelium, has tooth-inducing capacity, avoiding any issues that use of non-allogeneic cells may have for generation of nonessential organs such as teeth, in a clinical treatment.

## Maintaining the Odontogenic Potential In Vitro

### Three-Dimensional Microenvironmental Reprogramming

The arrangement of cells within a tissue plays an essential role in organogenesis, including tooth development [72]. In the condensed mesenchyme, during the tooth development, cells change their shape and size dynamically.

The size and shape of the condensed cell mass also dictate the final three-dimensional form of the organ, and abnormal condensation can result in developmental defects [73]. Mechanical stimuli can modulate cell lineage commitment and control development of various tissues during embryogenesis, and studies with cultured cells suggest that cell fate can be controlled mechanically by altering cell shape [74, 75]. These observations raise the possibility that physical alterations in cells that result from cell compaction in the condensed mesenchyme also could play an active role in the differentiation process [73].

In order to preserve the odontogenic signal in cells that have been expanded in vitro*,* Kuchler-Bopp et al. [72], have proposed a three-dimensional micro-culture system, where they tested the hanging drop method to study mixed epithelial-mesenchymal cell reorganization in a liquid medium, showing that the system offers the microenvironmental conditions for tooth histogenesis and organogenesis. It was shown that this method can provide control of the proportion and number of cells to be used, and the forming micro tissues showed homogeneous size.

### Cell Community Effect in Preserving the Odontogenic Potential

In 2016, Yang and colleagues [57••] proposed another approach to preserve the odontogenic signals in embryonic tooth germ cells that have rapidly lost their tooth-inducing capacity, once expanded in vitro. The study suggested that uncultured embryonic tooth germ mesenchymal cells were able to rescue cultured cells and enable them to fully participate into bioengineered tooth development, giving rise to dental pulp cells and odontoblasts. Although this rescue effect was not observed with postnatal dental pulp mesenchyme cells, this finding indicates that the presence of fresh (non-cultured) embryonic tooth germ cells can have a “community effect,” identified during embryonic development as a process that enables mixtures of different cells to differentiate along the same pathway.

## Conclusion

The newest trends in the dental research field propose a concept of regenerative dentistry, based on harnessing the natural healing abilities of the dental tissues through biological repair.

Although major steps are achieved in creating synthetic hierarchical materials that could be used in re-mineralization of the enamel, the enhanced functionality and the time for the re-mineralization of a tissue as the enamel remains an obstacle.

New data suggest that by mobilizing the stem cells through the Wnt signaling pathway—a particular cascade of molecules involved in cell-to-cell communication (essential for tissue repair and stem cell development), formation of reparative dentin can be achieved.

Moreover, usage of an already clinically tested drug known to stimulate Wnt signaling investigated in clinical trials for its potential to treat Alzheimer’s and other neurological disorders opens the possibility of a fast clinical translation for future biological dental treatments of dental cavities.

New research work contributed to the understanding of the underlying mechanisms of tooth development, mimicking the events in vitro*,* as an approach of bio-tooth engineering. The current focus of the researchers remains establishment of easily accessible cell sources that would maintain the odontogenic signal after in vitro expansion for future bioengineering.
